# The Implications of Tobacco Smoking on Acute Postoperative Pain: A Prospective Observational Study

**DOI:** 10.1155/2016/9432493

**Published:** 2016-03-29

**Authors:** Han-Liang Chiang, Yuan-Yi Chia, Huey-Shyan Lin, Chen-Hsiu Chen

**Affiliations:** ^1^Department of Anesthesiology, Kaohsiung Veterans General Hospital, Kaohsiung 813, Taiwan; ^2^School of Medicine, National Defense Medicine Center, Taipei 114, Taiwan; ^3^School of Nursing, Fooyin University, Kaohsiung 831, Taiwan; ^4^School of Medicine, National Yang-Ming University, Taipei 112, Taiwan

## Abstract

*Background*. The clinical importance of cigarette smoking on acute postoperative pain perception is not fully understood.* Methods*. To determine whether smokers who underwent major surgery need more postoperative opiate than do nonsmokers. We prospectively enrolled 407 male and 441 female participants who underwent in-hospital surgery. Current-smokers were compared with nonsmokers and past-smokers about opiate use during the first 72 h after surgery.* Results*. A greater proportion of males had more smoking history than females. The average age of male current-smokers is smaller than both nonsmokers and past-smokers. The surgical type (upper abdomen, lower abdomen, extremities, spine, and others) and duration of surgery have no differences between current-smokers, past-smokers, and nonsmokers. Statistically, the male current-smokers required more opiate analgesics during the first 72 h following surgery compared with the male nonsmokers and past-smokers; furthermore, the male current-smokers reported higher pain intensity when moving and at rest on day 1 after surgery.* Conclusions*. In this study, the male current-smokers required more morphine in the first 72 h after surgery than did the nonsmokers and past-smokers. Furthermore, smoking was more prevalent among the males than the females. Health care providers must be aware of the potential for increased narcotic requirements in male current-smokers.

## 1. Introduction

Nicotine markedly stimulates the central nervous system (CNS) and at low doses produces analgesia [1]. The antinociceptive effects of nicotine have been proven in experimental pain studies with both animal models and human models, and results have been independent of nicotine dependence [2–4]. This mechanism still is not fully understood [5].

Nicotine withdrawal in nicotine-dependent patients appears to result in hyperalgesia or lower pain threshold after surgical procedures [6]. Studies using electric shocks or exposure to freezing water indicated that nicotine-deprived smokers had a lower pain threshold than did smokers [7–9].

In most hospitals, smokers generally cannot smoke during hospitalization, which can induce nicotine withdrawal. Some studies have indicated that current-smokers or past-smokers who had nicotine withheld following surgery tended to use more opiate analgesics than did nonsmokers [10–12]. A retrospective clinical-based review of postoperative pain medications administered to coronary artery bypass graft patients showed that smokers required more pain medication than did nonsmokers after dose calculated from equivalent to the intramuscular morphine consumption during the first 48 h after surgery [12]. A small prospective study (13 patients in each group) demonstrated that smokers consumed more opioids by using patient-controlled analgesia (PCA) during the first 24 h after surgery than did nonsmokers [13]. However, other studies have suggested that these findings could be explained by demographic differences between tobacco users and nonusers [14]. The purpose of this current study is to assess a large cohort of surgical patients in a country where smoking is still widespread to assess potential associations between smoking status and postsurgical opioids analgesic use.

## 2. Materials and Methods

The protocol had been approved by the institution's IRB and all subjects provided informed consent prior to participating.

### 2.1. Study Overview

This is a prospective cohort study. Potential subjects were identified by systematically reviewing the surgical schedule for patients undergoing elective surgical procedures under general anesthesia. We did not know which patient would use patient-controlled analgesia (PCA) before surgery and collected data from postoperative PCA visiting chart. The first recruitment was in September 2010 and was closed in October 2012, and all patients were studied at Kaohsiung Veteran Hospital, Taiwan.

### 2.2. Participants

Inclusion criteria were candidates who have been scheduled to receive general anesthesia and using morphine PCA for pain control. Patients smoking at the time of interview and having regularly smoked for at least one year earlier were included in current-smokers group. Patients having smoked cigarettes >1 y prior to the interview were included in past-smokers group. Patients who had never smoked a cigarette were included in nonsmokers group. Past-smokers were asked about when they quit tobacco smoking. Patients were excluded if they were scheduled to undergo major thoracic cardiovascular surgery or if they had serious pulmonary disease, dementia, morbid obesity, liver disease, kidney disease, or American Society of Anesthesiologists Physical Status (ASA) above 4 or use chronic pain medications.

### 2.3. Anesthetic Management and Postoperative Pain Therapy

General anesthesia was induced and maintained by anesthesiologists in accordance with a standardized procedure. No premedication was given to the patients. After surgery, we would inform the patients on how to use the PCA and how to evaluate their pain intensity by using a visual analogue score ranging from 0 (*no pain*) to 10 (*worst possible pain*). The PCA was configured with the following initial settings: a 1 mg bolus of morphine upon patient demand, 5 min lockout time, and upper limit of 20 mg of morphine per 4 h. The dose of PCA was adjusted daily according to the patient's pain intensity while at rest. No nicotine was used as an analgesic during the perioperative period.

### 2.4. Data Abstracted

Information regarding confounders and demographic (age, sex, and smoking status), physiologic [hypertension, diabetes mellitus (DM)], anesthetic (ASA status, duration of anesthetics, and volume status), and surgical (surgical area) data were collected by standardized questionnaires and medical and anesthetic records. Information about history of pulmonary or cardiac disease was recorded from the patients' hospital admission history and physical examination findings. Tobacco smoking status or smoking dependence scale is determined through interviewer-assisted questionnaires (i.e., the Fagerstrom Test for Nicotine Dependence) 1 d before scheduled surgery. Pain intensity, the amount of PCA consumed, and related side effects were obtained from medical records by a trained research assistant during postoperative 72 h. The surgical site was derived from the operative report approximately 1 week after admission and classified as the following areas: (1) upper abdominal; (2) lower abdominal; (3) extremities; (4) spine; and (5) others. The type and duration of anesthesia were extracted from anesthetic records. In addition, all nonopiate and opiate analgesics used in the first 72 h following surgery were recorded. All the patients used a PCA device with morphine as the preferred method of analgesia.

### 2.5. Statistical Analysis

Continuous variables are described with mean and standard deviation; categorical variables are described with frequency and percentage. Independent samples one-way analysis of variance was employed to test difference among 3 tobacco smoking groups and Tukey multiple comparison technique was used to post hoc test for significant differences in the continuous variables [e.g., body mass index (BMI), visual analogue scales on movement (VASM), and postoperative morphine consumption (DOSE)]. Chi-square test or Fisher exact test (when appropriate) was conducted to identify any significant difference among 3 tobacco smoking groups in categorical variables (e.g., surgical area, DM, postoperative nausea, and vomiting). For male patients a multiple linear regression analysis using tobacco smoking status (two dummy variables: nonsmokers, past-smokers) and covariates (age, weight, and surgical duration) as independent variables and DOSE, VASM, and VASR as dependent variable, respectively, was performed to evaluate the relationship of tobacco smoking status with dependent variable after adjusting for potential confounders. Statistical analyses are performed using SPSS 22v, IBM, Armonk, NY. *P* values below 0.05 were considered statistically significant.

## 3. Results

**Figure 1:**
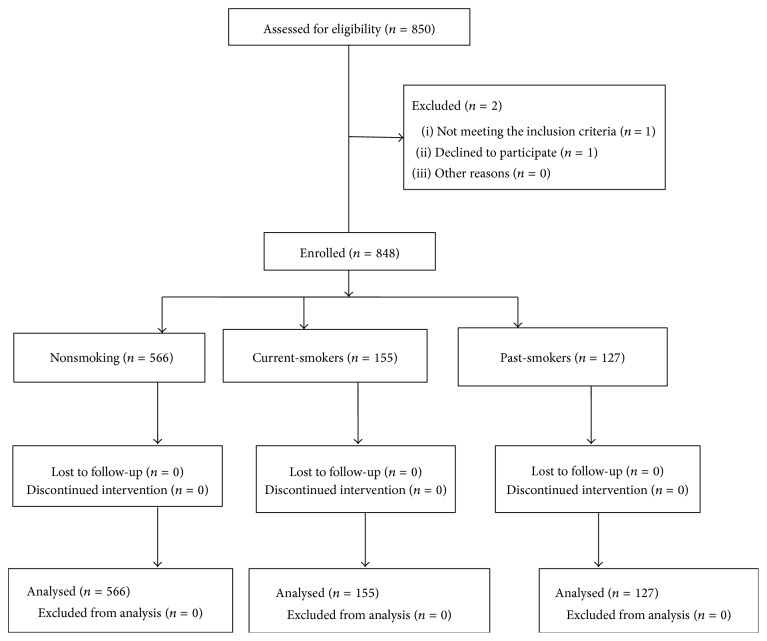
Patient eligibility and selection.

In total, 848 patients (566 nonsmokers, 155 current-smokers, and 127 past-smokers) were analysed (Figure 1). A greater proportion of males had a history of smoking than females (31.6% versus 5.9%), so we separated the male and female to conduct analysis. The demographic data by gender was shown in Tables 1 and 2, respectively. More male current-smokers and past-smokers had a history of drinking alcohol compared with the male nonsmokers, and the male current-smokers exhibited a higher rate of betel nut chewing than did the nonsmokers and past-smokers (Table 1). Surgical duration (hour) on male patients was presented in Table 3 and there were no significant differences among three groups. Statistically, the male current-smokers required more opiate analgesics than did the male nonsmokers and past-smokers during the 72 h postoperative period (nonsmokers versus current-smokers: *P* < 0.001; past-smokers versus current-smokers: *P* = 0.003) (Table 4). Then we used a multiple linear regression to compare adjusted mean differences across groups while controlling for relevant covariates (Table 4). Furthermore, the male current-smokers reported higher pain intensity when moving and at rest on day 1 after surgery. However, we do not have enough power to reflect finding among the female patients because of small size of current-smokers.

## 4. Discussion

The main finding of this study was that, statistically, the male current-smokers required more morphine than nonsmokers and past-smokers during the 72 h after surgery. The potential mechanisms of smoking and acute postoperative pain are complex and not fully understood [5]. Most of the studies are from the Western world where smoking has become increasingly taboo, thus presenting a selection bias. Our study has a unique Asian population where men still smoke at high rates, and this allows for better comparisons to be made between smokers and nonsmokers in regard to acute postoperative pain. In addition, previous work has failed to convincingly demonstrate that these differences are not just a feature of demographics. Creekmore et al. [12] observed that smokers used more opioids than nonsmokers after coronary arterial bypass graft (CABG) surgery. Demographic differences were noted between smokers and nonsmokers (young men smoke) but no attempt was made to adjust for these demographic differences. In this current study, we adjusted for age and eliminated sex (by not analysing female data) and examined noncardiac surgeries. Weingarten et al. published two observational studies on acute postoperative pain [14, 15]. One examined postoperative opioids requirements in CABG patients and adjusted for demographic factors. They also found that smokers used more opioids, but once adjusting for relevant demographic factors (age and sex) there was no difference in opioids use. Another examined postoperative opioids requirements in patients undergoing bariatric surgery (mostly female) and also found that smokers used more opioids, but once adjusting for relevant demographic factors (age and sex) there was no difference in opioids use. Unlike the Weingarten studies, we examined different surgeries, eliminated sex as a variable (an important variable for postoperative opioid use), and examined a population with a high prevalence of smoking.

On the other side, some studies showed that smokers experience higher levels of pain and suffer from severe pain more frequently following surgery. One prospectively collected pre-, intra-, and postoperative data from 2157 adults who underwent elective ambulatory surgery under general anesthesia from 12 United States hospitals [16]. They had larger number of patients involved but did not adjust for cofounders and only observed VAS score after surgery but not analgesic consumption. Another study published in 2014 [17] evaluated postoperative meperidine demand between smoking and nonsmoking groups. However, factors other than smoking status that are known to influence postoperative opioid use, such as age, sex, and surgical characteristics, may not be well controlled.

Because there are smoke-free policies in most health care facilities, it is unknown whether smokers deprived of cigarettes require more postoperative analgesia in physiological basis and contribute to our finding. Warner [18] indicated nicotine withdrawal scores suggested that smokers did not consistently experience withdrawal symptoms in the immediate postoperative period, including patients highly dependent on nicotine preoperatively. Nicotine administration in postoperative smokers has not demonstrated an improvement in postoperative pain. One study conducted in Turkey [19] that included 85 subjects (about 60% were smokers) revealed perioperative administration of a high-dose transdermal nicotine did not improve postoperative pain control or decrease the analgesic requirement in pelvic gynecological surgery. Another study also made a conclusion that transdermal nicotine, 5–15 mg, failed to relieve postoperative pain or reduce opioids use in smokers [20].

Age is another important confounding factor in our study. We found that the age of smokers was significantly lower than the two other groups (*P* < 0.001). In an article published in 1996 entitled “Age Is the Best Predictor of Postoperative Morphine Requirements” [21], the indicated confounding factors included were age, weight, operative site, verbal numeric pain score (at rest and on movement), and a nausea/vomiting score. In a subgroup of 78 of these patients, although the interpatient variability in PCA morphine doses was large (differences of up to 10-fold in each age group), the best predictor of PCA morphine requirement in the first 24 h after surgery (the amount required in the 24 h after the initial loading dose) was the age of the patient. In our multiple regression model, we still found significant total morphine requirement differences between smokers and the other two groups after adjusting for age, weight, and surgical duration.

Additionally, our country is conservative with opioids administration and very few subjects were tolerant to opioids preoperatively. On the other hand, a high percentage of male current-smokers also drink alcohol and consume betel nut regularly. Patients that drink alcohol and consume betel nuts have poorer health behaviors and may have poorer response to stress, which could influence postoperative pain [22].

## 5. Study Limitations

1. We had inadequate number of women smokers (about 5%) to perform a meaningful analysis. Most studies on smoker-associated analgesia have mostly included only men; those that have included women have produced mixed findings [23, 24]. A previous study on a smoking cessation clinic in Taiwan [25] indicated that men account for a markedly higher proportion of smokers than females (47% versus 4%). Hence, because female smokers are difficult to recruit, research results related to them often do not reach the level of statistical significance.
2. This study did not conduct a double-blinded experiment, and the participants might have known how they were grouped.

## 6. Conclusion

In this study, we recruited 848 patients who underwent elective surgery under general anesthesia. To explore the relationship of smoking status and patient-control analgesia consumption, we separated them into 3 groups (nonsmokers, current-smokers, and past-smokers). We found the male current-smokers required more morphine in the first 72 h after surgery than did the nonsmokers and past-smokers. Furthermore, smoking was more prevalent among the males than the females. Compared with the nonsmokers, more current-smokers and past-smokers had a history of drinking alcohol. Health care providers must be aware of the potential for increased narcotic requirements of male current-smokers.

## Figures and Tables

**Table 1:** Demographic data on male patients.

Variable	Nonsmokers	Current-smokers	Past-smokers	*P* value
Age (y/o)	63.5 ± 15.4	54.5 ± 14.3^*∗*^	66.4 ± 14.2	<0.001
Height (cm)	165.4 ± 10.2	167.2 ± 6.6	165.6 ± 11.0	0.601
Weight (kg)	69.3 ± 13.7	67.6 ± 12.1	67.9 ± 12.3	0.46
ASA (I/II/III)	7/98/51	5/83/41	1/60/62	0.078
BMI (kg/m^2^)	24.8 ± 3.4	24.1 ± 3.7	24.6 ± 4.0	0.257
Intraoperative fluid infusion (mL)	2977 ± 1792	3158 ± 2079	2996 ± 1912	0.168
Intraoperative blood loss (mL)	494 ± 410	526 ± 487	480 ± 468	0.704
Intraoperative urine output (mL)	521 ± 487	782 ± 686	626 ± 607	0.143
Smoking dependence scale	—	3.9 ± 2.3	—	—
Duration after quitting smoking (year)	—	—	11.5 ± 4.0	—

Surgical area				
Upper abdomen	21 (13%)	10 (8%)	22 (18%)	0.190
Lower abdomen	96 (61%)	62 (48%)	74 (60%)	
Extremities	21 (13%)	16 (12%)	13 (11%)	
Spine	15 (10%)	17 (13%)	6 (5%)	
Others	3 (2%)	24 (19%)	8 (6%)	

Past history				
HTN	66 (42%)	46 (36%)	51 (42%)	0.115
DM	29 (18%)	19 (15%)	27 (22%)	0.279
Alcohol drinking	15 (10%)^†^	28 (22%)	27 (22%)	0.007
Betel nut chewing	3 (2%)	25 (19%)^*∗*^	11 (9%)	<0.001

Case number	156	129	123	Total cases: 408

Data were presented as mean ± SD or number (%). BMI: body mass index; HTN: hypertension; DM: diabetes mellitus. One-way ANOVA *∗*: *P* < 0.05 when compared with the groups of nonsmokers and past-smokers. †: *P* < 0.05 when compared with the group of current-smokers or past-smokers.

**Table 2:** Demographic data on female patients.

Variable	Nonsmokers	Current-smokers	Past-smokers	*P* value
Age (y/o)	58.8 ± 1.2	53.5 ± 10.7^*∗*^	62.8 ± 13.4	<0.001
Height (cm)	154.5 ± 6.3	158.3 ± 7.6	150.5 ± 5.0	0.132
Weight (kg)	60.1 ± 11.9	63.1 ± 15.6	62.1 ± 8.3	0.437
ASA (I/II/III)	39/256/114	3/16/7	0/3/3	0.787
BMI (kg/m^2^)	25.2 ± 4.9	25.2 ± 6.1	27.8 ± 3.6	0.435
Intraoperative fluid infusion (mL)	2801 ± 1588	3055 ± 1350	2241 ± 993	0.239
Intraoperative blood loss (mL)	421 ± 407	520 ± 471	425 ± 288	0.791
Intraoperative urine output (mL)	619 ± 525	629 ± 539	516 ± 327	0.371
Smoking dependence scale	—	2.8 ± 2.1	—	—
Duration after quitting smoking (year)	—	—	10.1 ± 3.6	—

Surgical area				
Upper abdomen	54	1	0	0.506
Lower abdomen	237	16	5	
Four limbs	61	4	1	
Spine	56	5	0	
Others	1	1	0	

Past history				
HTN	156 (38%)	6 (23%)	2 (33%)	0.311
DM	74 (18%)	3 (12%)	2 (33%)	0.371
Alcohol drinking	4 (1%)	4 (15%)^†^	0 (0%)	0.001^*∗*^
Betel nut chewing	1 (0.3%)	1 (4%)	0 (0%)	0.140

Case number	409	26	6	Total cases: 441

Data were presented as mean ± SD or number (%). BMI: body mass index; HTN: hypertension; DM: diabetes mellitus. One-way ANOVA *∗*: *P* < 0.05 when compared with the groups of nonsmokers or past-smokers.†: *P* < 0.05 when compared with the group of nonsmokers or past-smokers.

**Table 3:** Surgical duration hour in different surgical areas between three male groups.

	Nonsmoker	Smokers	Past-smokers	*P* value
Upper abdomen	5.1 ± 3.4	5.8 ± 3.0	5.8 ± 5.0	0.8
Lower abdomen	4.9 ± 2.1	5.2 ± 2.5	5.0 ± 2.1	0.69
Extremities	3.3 ± 1.9	4.0 ± 3.4	4.1 ± 3.8	0.66
Spine	8.3 ± 3.2	6.7 ± 2.6	6.6 ± 2.2	0.23
Others	13.7 ± 1.8	11.5 ± 3.3	9.3 ± 4.1	0.11

Data were presented as mean ± SD or number (%).

**Table 4:** Comparisons for postoperative outcomes on male patients.

		Nonsmokers	Current-smokers	Past-smokers	*P* value	Adjusted *P* value^a^	
						Gr1 versus Gr2	Gr2 versus Gr3
POD1	VASM1	5.1 ± 1.8	5.6 ± 1.8^*∗*^	5.0 ± 1.8	0.044	0.090	0.096
	VASR1	2.3 ± 1.2	2.9 ± 1.6^*∗*^	2.2 ± 1.0	0.006	0.045	0.208
	DOSE1 (mg)	25.7 ± 15.0	32.2 ± 19.8^*∗*^	25.9 ± 16.7	0.004	0.011	0.056
	PONV1	24 (15.4%)	23 (17.8%)	11 (8.9%)	0.113		

POD2	VASM2	4.1 ± 1.5	4.4 ± 1.5	4.1 ± 1.7	0.179	0.158	0.344
	VASR2	1.4 ± 1.1	1.8 ± 1.3	1.6 ± 1.0	0.062	0.127	0.959
	DOSE2 (mg)	45.3 ± 25.9	64.7 ± 38.5^*∗*^	46.8 ± 28.1	<0.001	<0.001	<0.001
	PONV2	12 (7.6%)	5 (3.9%)	3 (2.5%)	0.106		

POD3	VASM3	3.1 ± 1.3	3.4 ± 1.2	3.1 ± 1.2	0.187	0.818	0.818
	VASR3	0.9 ± 0.6	1.2 ± 1.0	0.9 ± 0.8	0.185	0.584	0.927
	DOSE3 (mg)	55.7 ± 32.9	77.9 ± 49.0^*∗*^	55.7 ± 33.3	<0.001	<0.001	0.002
	PONV3	4 (2.5%)	3 (2.3%)	0 (0%)	0.213		

	Satisfied score	8.4 ± 0.8	8.3 ± 1.0	8.3 ± 0.9	0.833		
	Morbidity	20 (13%)	21 (16%)	20 (17%)	0.649		

Data were presented as mean ± SD or number (%). Gr1: nonsmokers group; Gr2: current-smokers group; Gr3: past-smokers group; POD1: postoperative day one; POD2: postoperative day two; POD3: postoperative day three; PONV: postoperative nausea and vomiting; VASM: visual analogue scales on movement; VASR: visual analogue scales at rest; DOSE: postoperative morphine consumption; *∗*: *P* < 0.05 when compared with nonsmokers or past-smokers. ^a^Controlling for age, weight, and surgical duration when dependent variable is DOSE; controlling for age and surgical duration when dependent variable is VASM and VASR.

## Additional Points

This report describes human research. IRB contact information is Kaohsiung Veterans General Hospital, Kaohsiung, Taiwan.
